# Ryanodine-induced vasoconstriction of the gerbil spiral modiolar artery depends on the Ca^2+^ sensitivity but not on Ca^2+^ sparks or BK channels

**DOI:** 10.1186/s12899-016-0026-z

**Published:** 2016-11-02

**Authors:** Gayathri Krishnamoorthy, Katrin Reimann, Philine Wangemann

**Affiliations:** 1 Anatomy & Physiology Department, Cell Physiology Laboratory, Kansas State University, 228 Coles Hall, Manhattan, Kansas 66506-5802 USA; 2 Department of Otolaryngology–Head and Neck Surgery, Tübingen Hearing Research Centre, and Molecular Physiology of Hearing, University of Tübingen, Tübingen, Germany

**Keywords:** Ca^2+^ spark, Ca^2+^ sensitivity, Spiral modiolar artery, Ryanodine, Vascular diameter, BK channels, Tetracaine

## Abstract

**Background:**

In many vascular smooth muscle cells (SMCs), ryanodine receptor-mediated Ca^2+^ sparks activate large-conductance Ca^2+^-activated K^+^ (BK) channels leading to lowered SMC [Ca^2+^]_i_ and vasodilation. Here we investigated whether Ca^2+^ sparks regulate SMC global [Ca^2+^]_i_ and diameter in the spiral modiolar artery (SMA) by activating BK channels.

**Methods:**

SMAs were isolated from adult female gerbils, loaded with the Ca^2+^-sensitive flourescent dye fluo-4 and pressurized using a concentric double-pipette system. Ca^2+^ signals and vascular diameter changes were recorded using a laser-scanning confocal imaging system. Effects of various pharmacological agents on Ca^2+^ signals and vascular diameter were analyzed.

**Results:**

Ca^2+^ sparks and waves were observed in pressurized SMAs. Inhibition of Ca^2+^ sparks with ryanodine increased global Ca^2+^ and constricted SMA at 40 cmH_2_O but inhibition of Ca^2+^ sparks with tetracaine or inhibition of BK channels with iberiotoxin at 40 cmH_2_O did not produce a similar effect. The ryanodine-induced vasoconstriction observed at 40 cmH_2_O was abolished at 60 cmH_2_O, consistent with a greater Ca^2+^-sensitivity of constriction at 40 cmH_2_O than at 60 cmH_2_O. When the Ca^2+^-sensitivity of the SMA was increased by prior application of 1 nM endothelin-1, ryanodine induced a robust vasoconstriction at 60 cmH_2_O.

**Conclusions:**

The results suggest that Ca^2+^ sparks, while present, do not regulate vascular diameter in the SMA by activating BK channels and that the regulation of vascular diameter in the SMA is determined by the Ca^2+^-sensitivity of constriction.

## Background

Cochlear function is sensitive to dynamic changes in cochlear blood flow that is responsible for the delivery of oxygen and glucose and the removal of CO_2_ [1, 2]. Regulation of cochlear blood flow is essential for hearing and is important as a treatment strategy for the restoration of hearing loss in humans [3–8]. Homeostatic regulation of blood flow in the cochlear capillary beds is achieved by the dynamic adjustment of the vascular diameter or “tone” of pre-capillary arteries and arterioles against systemic changes of pressure, nerve and metabolic activity [9–14]. The mechanisms involved in such regulation of the spiral modiolar artery, the principal artery of the cochlear blood supply, remain to be elucidated.

Smooth muscle cells of most arteries exhibit “Ca^2+^ sparks”, which are transient local elevations of Ca^2+^ caused by the opening of ryanodine receptors (RyRs) in the sarcoplasmic reticulum (SR) [15, 16]. In most smooth muscle cells, Ca^2+^ sparks activate BK channels, leading to membrane hyperpolarization, reduced activity of l-type voltage-dependent Ca^2+^ channels (VDCCs), decrease in [Ca^2+^]_i_ and smooth muscle relaxation [15, 17–19]. Thus, the triad of Ca^2+^ sparks, VDCCs and BK channels effectively regulates intracellular Ca^2+^ to oppose vasoconstriction and maintain blood flow to the underlying tissue. Activation of BK channels by Ca^2+^ sparks is a potent vasodilatory mechanism to regulate SMC global [Ca^2+^]_i_ and vascular diameter and a prominent feature in blood vessels of the cerebral, kidney, mesenteric and cardiac microcirculation [17–21].

We have recently demonstrated Ca^2+^ sparks in smooth muscle cells of the intact SMA [22]. In this study, we investigate whether Ca^2+^ sparks regulate the global Ca^2+^ and vascular diameter in the SMA. Our results demonstrate that Ca^2+^ sparks are also present in the pressurized SMA but do not regulate vasodilation of the SMA by activating BK channels. Instead, the effects produced by ryanodine, which eliminates Ca^2+^ sparks, are dictated by the pressure-dependent changes in the Ca^2+^ sensitivity of contraction.

## Methods

### Ethics statement

All procedures involving animals were approved by the Institutional Animal Care and Use Committee at Kansas State University (IACUC#: 2961 and 3245).

### Isolation of the spiral modiolar artery (SMA)

Female gerbils between the ages of 4 to 12 weeks (Charles River, Wilmington, MA) were anesthetized with tri-bromo-ethanol (560 mg/kg i.p.) and sacrificed by decapitation. Auditory bullae were harvested and the spiral modiolar arteries (SMAs) separated from the cochlea by microdissection in HEPES-buffered physiological saline solution (PSS) at 4 C°.

### Pressurization and superfusion

Segments of the SMA were pressurized and perfused in a custom-built bath chamber using a variable hydrostatic pressure column connected to a motorized set of concentric glass pipettes (Wangemann Instruments, Kansas State University, KS) mounted on an inverted microscope (Axiovert 200, Carl Zeiss, Göttingen, Germany) [12]. Briefly, arteries were held by a holding pipette and luminally perfused with a perfusion pipette at one end while the other end was occluded using a blunt glass pipette. All pipettes were prepared using a custom-built micro-forge. The pressurized vessel was superfused in the bath with either HEPES-buffered PSS at a rate of 1.6 ml/min, permitting one complete exchange of the bath volume (~70 μl) within ~3 s. Experiments were conducted at 37 °C. Bath temperature was maintained by a triple heating system consisting of regulating the temperatures of the superfusate (8-line heater, CL-100, Warner Instruments, Hamden, CT, USA), the bath chamber (TC 324B, Warner Instruments) and the microscope objective (TC 324B, Warner Instruments).

### Measurements of cytosolic Ca^2+^ signals

Cytosolic Ca^2+^ signals in SMCs were monitored as spatial and temporal changes in the fluorescence intensity of the indicator dye fluo4. For loading the dye, pressurized vessel segments were incubated in 2.5 μM fluo4-AM (Invitrogen, Carlsbad, CA, USA) for 15 min at 37 °C, followed by wash and superfusion with HEPES-buffered PSS. The dye loaded virtually exclusively into SMCs. Fluo4 was excited by a 488 nm argon laser. Fluorescence emissions were filtered by a 488 notch and two long-pass filters (490 nm and 505 nm) and recorded by a photomultiplier through an open pinhole (LSM 510 Meta, Carl Zeiss).

### Ca^2+^ sparks

Ca^2+^ sparks in SMCs of pressurized SMA were detected in frame scans and line scans. For frame scans, tangential images of the vascular wall (32.14 μm × 10.04 μm) were recorded using an oil-immersion objective (Plan-Neofluar 40× 1.3 N.A., Carl Zeiss) at a temporal resolution of ~61 images/s (16.3 ms/frame) and a spatial resolution of 0.25 μm × 0.25 μm per pixel. Spark sites were identified using custom-designed software, SparkAn, developed by Dr. Adrian D. Bonev (University of Vermont, VT, USA) in IDL 5.0.2 (Research Systems, Boulder, CO) and kindly provided for use by Dr. Adrian D. Bonev and Dr. Mark T. Nelson (University of Vermont). Ca^2+^ sparks were detected by dividing an area spanning 2.01 μm (8 pixels) × 2.01 μm (8 pixels) in each frame by a baseline (F_0_) that was obtained by averaging 10 frames without Ca^2+^ spark activity. Spark traces and 2-dimensional pseudo-color images were obtained as F/F_0_. 3-dimensional ratio images were generated by SparkAn.

Line-scan recordings of 5 s duration each were performed at a Ca^2+^ spark site to determine the temporal parameters of Ca^2+^ sparks in SMCs. Lines (0.15 μm × 12.4 μm) were recorded using an oil-immersion objective (Plan-Neofluar 40× 1.3 N.A.) at a temporal resolution of ~521 lines per second (0.82 ms per line). For spark measurements at different pressures, three 5 s line-scans were performed first at 60 cmH_2_O, followed by three line-scans at 40 cmH_2_O, followed by three line scans at 60 cmH_2_O. Time intervals between consecutive line-scans were 15 s to allow for recovery. Time intervals between pressure-changes were 45 s. For experiments in ryanodine and tetracaine, 10 μM ryanodine or 100 μM tetracaine was introduced after the third line-scan in PSS and scans were resumed after 2 min. Recordings were analyzed as described earlier [11]. For presentation, a single 5 s line-scan image was contrast-enhanced to highlight the occurrence of Ca^2+^ spark events.

### Determination of length and height of smooth muscle cells

To calculate Ca^2+^ spark density, cell length and cell height of single SMCs were estimated from scanned images of pressurized SMA loaded with BCECF (Sigma-Aldrich). SMC length and height were determined to be 132 ± 17 μm and 3.2 ± 0.1 μm based on images of 9 vessels that each covered 20 – 30 cells. These values correspond to a cell volume of ~1 pl, which is consistent with SMCs from other vessels [15].

### Simultaneous measurements of vascular diameter and global cytosolic Ca^2+^

Pressurized vessels (40 or 60 cmH_2_O), loaded with the indicator dye fluo4 as described above, were superfused with HEPES-buffered PSS. To record the inner diameter of pressurized vessels simultaneously with changes in the global cytosolic Ca^2+^ in SMCs, images (225 μm × 225 μm) were recorded using an oil-immersion objective (Plan-Neofluar 40× 1.3 N.A.) with a temporal resolution of 1 image/s (983 ms/frame) and a spatial resolution of 0.44 μm × 0.44 μm per pixel. In addition to filtering and recording fluorescence emissions as described above, the transmitted light from the argon laser was detected by a second photomultiplier. Inner diameter was measured by automatic edge detection by a method developed and described in Reimann et al. 2011 [12]. Inner diameter (ID) was detected from acquired real time transmitted light images, using a custom written data acquisition program (﻿Dr. W. Gil Wier, University of Maryland). Edge detection data were analyzed using a custom written analysis program in Origin 6.0 (﻿Dr. P. Wangemann, Kansas State University). Inner diameter changes were normalized against the average of 30 data points obtained in PSS at the beginning of the experiment (basal vascular tone). Fluorescence intensity values from 5–10 SMCs per pressurized vessel were averaged and normalized between the fluorescence values in Ca^2+^ free solution and the fluorescence value in PSS before the addition of drugs according to the formula Norm Ca^2+^ = (([Ca^2+^]_i_ ‐ [Ca^2+^]_0_)/([Ca^2+^]_PSS_ ‐ [Ca^2+^]_0_)) + 1, where [Ca^2+^]_0_ is marked *b* and [Ca^2+^]_PSS_ is marked in Figs. 2b, 3b, 3e, 4b, 5b, and 7b. *a*

### Ca^2+^ sensitivity

Simultaneous measurements of diameter and global cytosolic Ca^2+^ were performed to determine the Ca^2+^-sensitivity of constrictions. In these experiments, following equilibration in HEPES-buffered PSS for 15 min, arteries were superfused with saline solutions containing 0, 1, 3 and 10 mM Ca^2+^ in 2 min steps, first at 60 cmH_2_O followed by the same protocol at 40 cmH_2_O. Data points for concentration curves were obtained by averaging diameter and fluorescence intensity measurements over the last 30s of each Ca^2+^ step and normalizing against the average value obtained in PSS at 60 cmH_2_O. Data points from individual vessels were fitted to a modified Hill equation:

$$ Dia= Base+\frac{\left( Max- Base\right)\times {\left[C{a}^{2+}\right]}^h}{\left({\left[F{C}_{50}\right]}^h+{\left[C{a}^{2+}\right]}^h\right)} $$

where *Dia* is the normalized diameter, *Max* is the diameter at 60 cmH_2_O in PSS containing 1 mM Ca^2+^, *Base* is the maximum achievable constriction with respect to *Max* for the female gerbil SMA estimated from previous observations [23], [Ca^*2+*^] is the normalized cytosolic Ca^2+^ concentration, *h* is the slope coefficient, and *FC*_*50*_ is the fold-change in the global cytosolic Ca^2+^ concentration that is necessary for a half-maximal constriction. The slope coefficient *h* was set to -5.2 and *Max* was clamped to 100 %. Two *FC*_*50*_ values, one each for 60 cmH_2_O and 40 cmH_2_O, were obtained for each experiment. For presentation, normalized Ca^2+^ and diameter data were averaged and fitted with the equation above using average *FC*_*50*_ values.

### Solutions and drugs

HEPES-buffered PSS contained (in mM): 150 NaCl, 5 HEPES, 3.6 KCl, 1 MgCl_2_, 1 CaCl_2_ and 5 glucose, pH adjusted to 7.4 at 37 °C. Ca^2+^-free solutions were devoid of CaCl_2_ and contained 1 mM EGTA. 100 μM papaverine hydrochloride (Pap) was added to the Ca^2+^ free solution wherever indicated. Stock solutions of ryanodine (Ryn, 20 mM, Enzo Life Sciences, NY or Santa Cruz Biotechnologies, Santa Cruz, CA), tetracaine (Tet, 100 mM, Sigma- Aldrich), paxilline (Pax, 10 mM, Sigma-Aldrich) and papaverine (Pap, 250 mM, Sigma-Aldrich) were prepared in DMSO and stored at -20 °C and freshly diluted to target concentration in solution when required taking care that the final DMSO concentration in solution did not exceed 0.1 %. Endothelin-1 (ET-1, 1 μM, Sigma-Aldrich) and iberiotoxin (IbTx, 2 μM, Alomone Labs, Jerusalem, Israel) were always freshly prepared in PSS for immediate use.

## Results

### Ca^2+^ sparks in the pressurized spiral modiolar artery

**Fig. 1:**
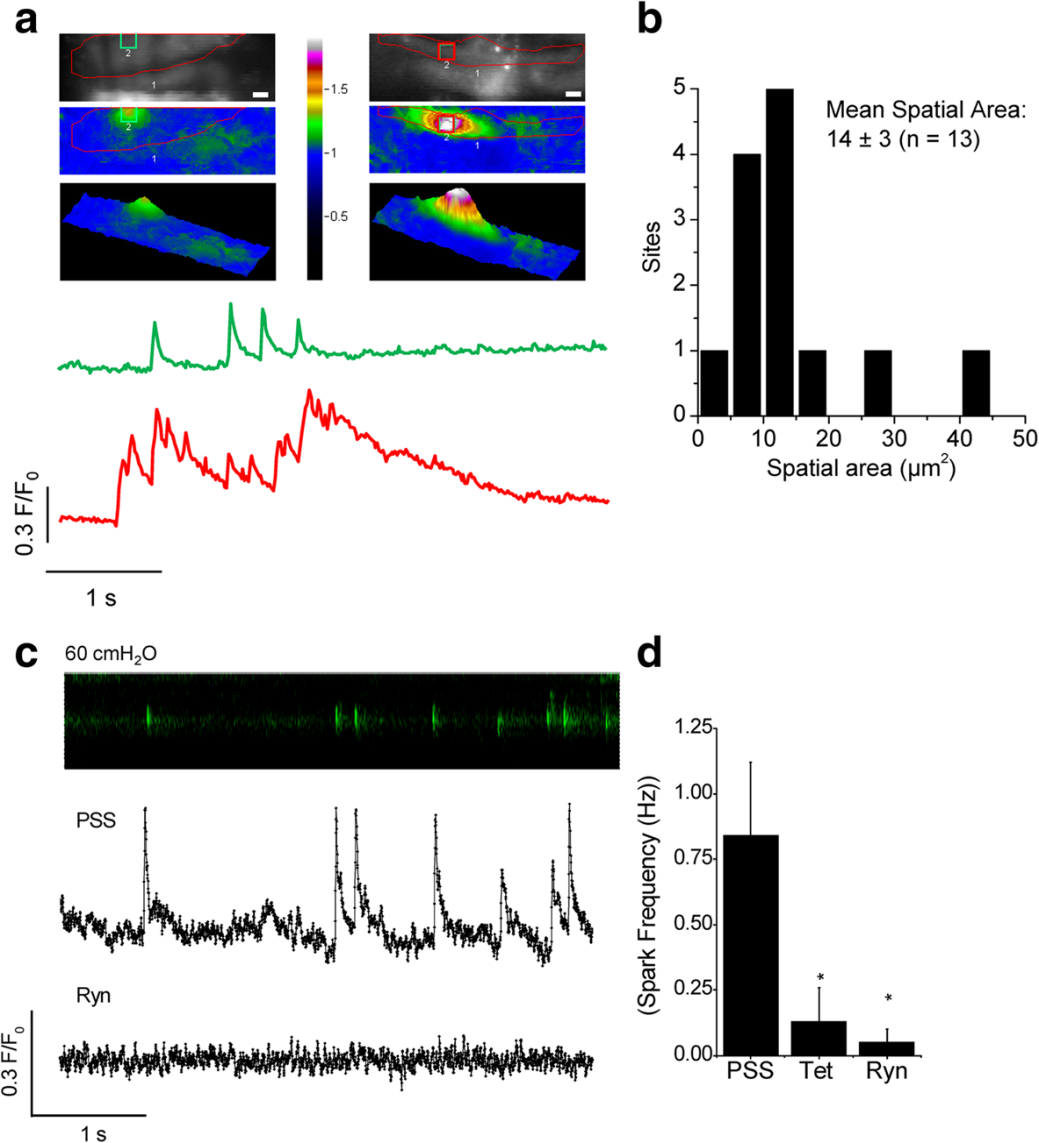
Ca^2+^ sparks in smooth muscle cells of the pressurized spiral modiolar artery. **a** Ca^2+^ spark site captured during a 5 s frame scan of SMCs in a pressurized (60 cmH_2_O) SMA. Grey panel (*top panels*) depicts the average of 10 frames that do not contain a Ca^2+^ spark. Outline (*in red*) depicts the visible portion of the cell. Scale bar = 2 μm. Pseudo-color image depicts an average (*left middle panel*) and a large (*right middle panel*) Ca^2+^ spark at its peak. Regions of interest (ROIs) are 2 μm × 2 μm. (*Bottom panels*) show a 3D rendering of the 2D images. Traces (*green* and *red*) represent the fluorescence intensity changes occurring at the corresponding ROIs shown in the panels above. **b** Histogram showing the distribution of the calculated spatial area of Ca^2+^ spark sites. **c** Contrast-adjusted 5 s line-scan recording of a Ca^2+^ spark site in a SMC of a pressurized (60 cmH_2_O) SMA and the corresponding fluorescence intensity traces recorded in PSS and in the presence of 10 μM ryanodine (Ryn). **d** Spark frequency in PSS (*n* = 9), 100 μM tetracaine (Tet,* n* = 3) and 10 μM ryanodine (Ryn, *n* = 4)

**Fig. 2:**
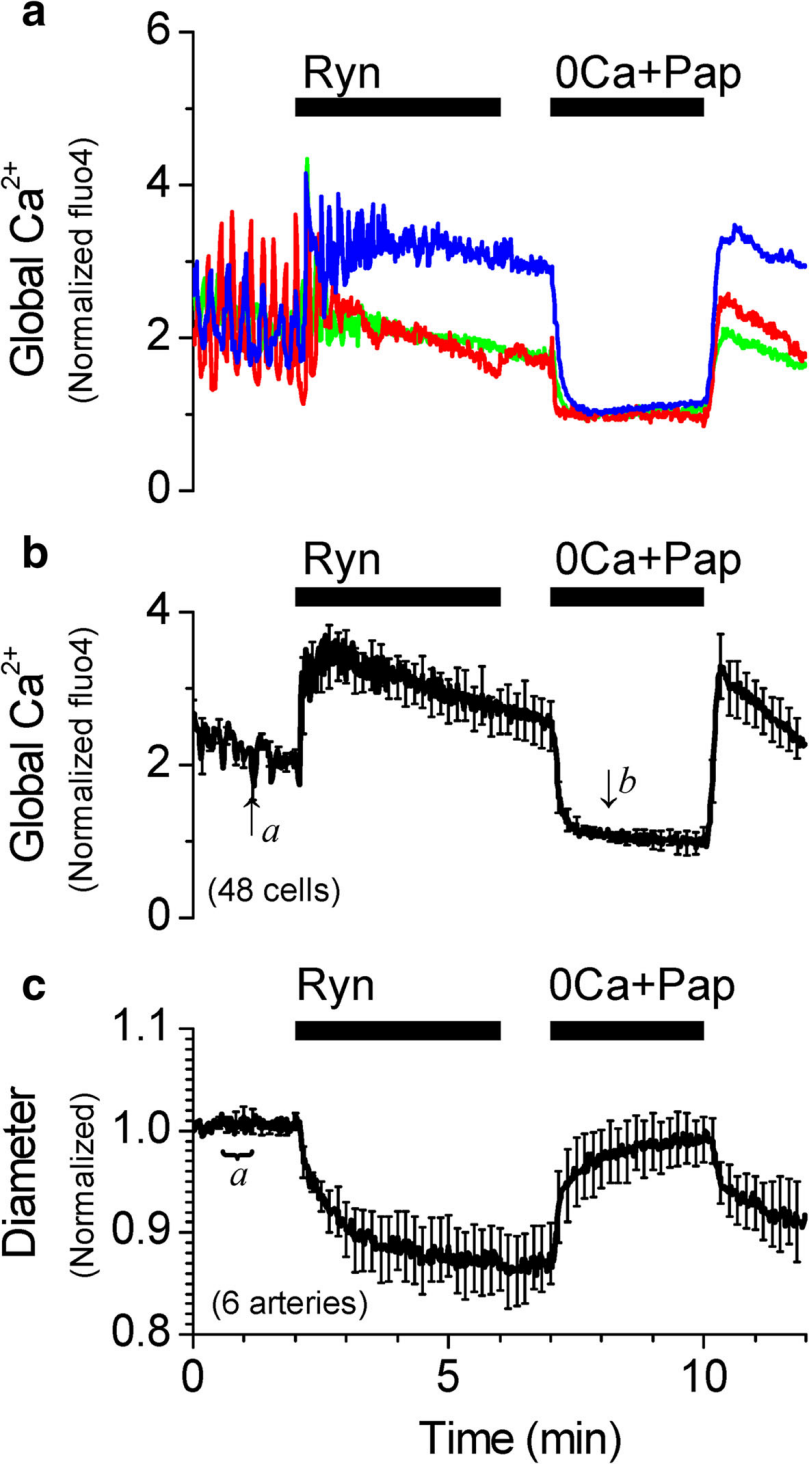
Inhibition of Ca^2+^ sparks with ryanodine increases the global [Ca^2+^]_i_ and constricts the SMA. **a** Representative recordings of [Ca^2+^]_i_ changes from single smooth muscle cells from a pressurized (40 cmH_2_O) SMA in response to 10 μM ryanodine (Ryn). **b** Average of normalized traces of [Ca^2+^]_i_ changes at 40 cmH_2_O (48 cells). Traces in **a** and **b** were normalized as described in Methods. **c** Average trace of corresponding changes in vascular diameter (6 arteries). Diameter changes were normalized to the average of values recorded between 30–60 s (average value indicated by ‘a’ was set to 1). [Ca^2+^]_i_ and diameter data were simultaneously acquired at 1 s intervals, however, for clarity, error bars (sem) are plotted only every 10 s

Ca^2+^ sparks and waves were recently reported in the intact unpressurized gerbil SMA [22]. We now report Ca^2+^ sparks and Ca^2+^ waves in SMCs of the pressurized gerbil SMA (Fig. 1). Ca^2+^ spark sites were consistently observed in frame scans (Fig. 1a), with super Ca^2+^ sparks, of larger cross-sectional area and longer duration of elevated Ca^2+^ observed occasionally, which may reflect the combined synchronized activity of two or more closely spaced Ca^2+^ sparks sites. The average spatial area of spark sites was 14 ± 3 μm^2^, corresponding to a spatial width of ~4 μm. Two out of 13 recorded Ca^2+^ spark sites had larger spatial areas between 25 – 50 μm^2^ (Fig. 1b). An average of 1.3 ± 0.2 Ca^2+^ spark sites were present in 200 ± 10 μm^2^ of recorded area (*n* = 10), corresponding to ~47 % of one cell, giving a spark density of 2.8 ± 0.3 spark sites/cell. The frequency of Ca^2+^ spark occurrence per site and other temporal parameters were measured in pressurized SMA using line-scans (Fig. 1c). Spark frequency per site increased from 0.6 Hz to 0.9 Hz, as pressure increased from 40 cmH_2_O to 60 cmH_2_O (Table 1). However, increasing pressure from 40 to 60 cmH_2_O did not alter the spark amplitude, the rise-time or the half-time of decay (Table 1). Ca^2+^ sparks were completely eliminated in the presence of 10 μM ryanodine (Fig. 1c) and significantly decreased in frequency in the presence of 100 μM tetracaine (Fig. 1d). Ca^2+^ oscillations exhibiting wave-like phenomena were also observed in SMCs and were likewise abolished by application of 10 μM ryanodine (Fig. 2a).

**Table 1:** Parameters of Ca^2+^ sparks

Pressure (cmH_2_O)	Frequency per site (Hz)	Amplitude (F/F_0_)	Rise-Time (ms)	Half-decay-time (ms)
40	0.6 ± 0.1 (*n* = 17)	1.53 ± 0.03 (*n* = 65)	18.3 ± 0.7 (*n* = 65)	19.8 ± 1.2 (*n* = 64)
60	^a^ 0.9 ± 0.1 (*n* = 17)	1.49 ± 0.03 (*n* = 110)	16.9 ± 0.3 (*n* = 110)	17.8 ± 0.8 (*n* = 110)

^a^ indicates significance between parameter values measured at 40 and 60 cmH_2_O

### Effects of inhibitors of Ca^2+^ sparks and BK channels on global Ca^2+^ and vascular diameter of the SMA

**Fig. 3:**
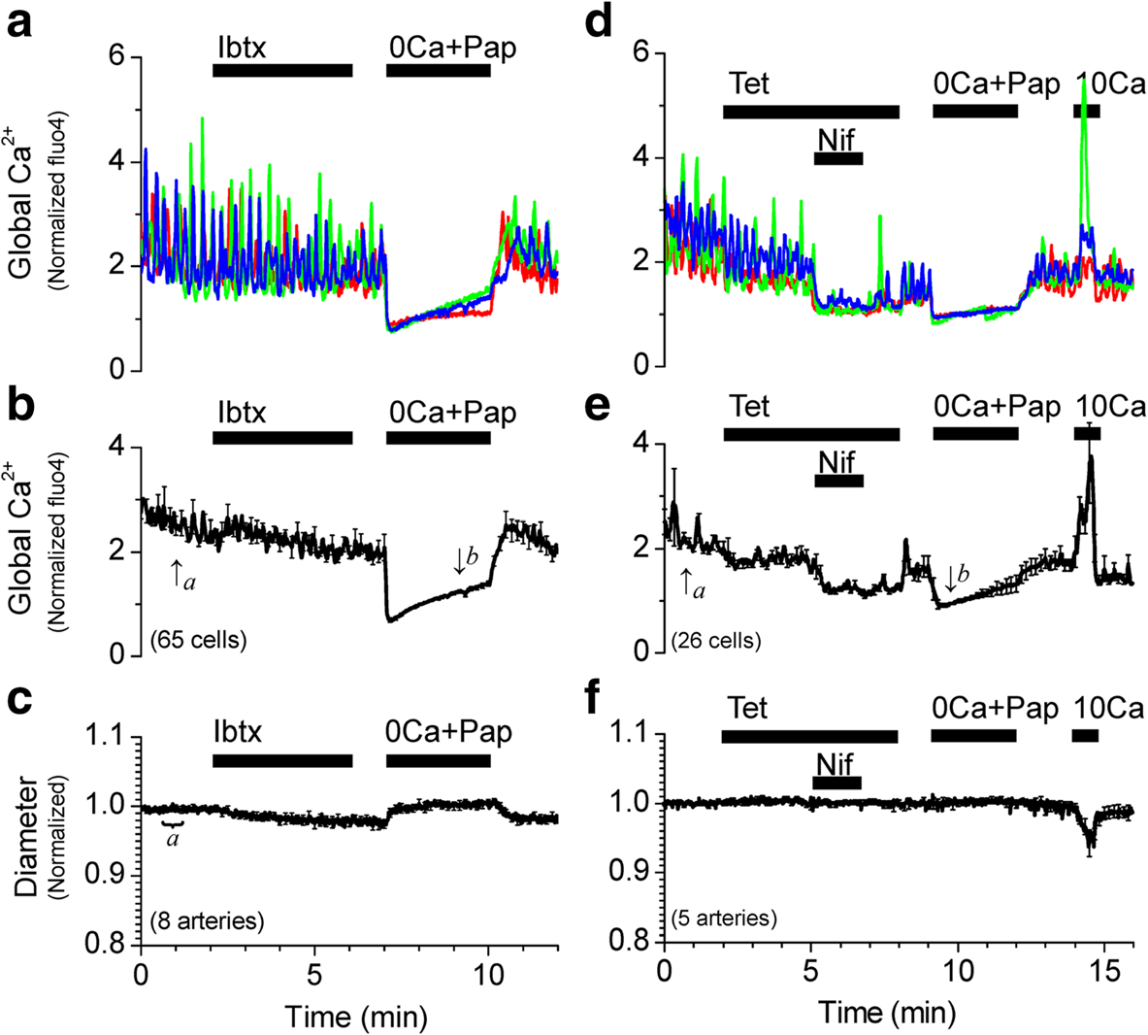
Inhibition of BK channels with iberiotoxin or inhibition of Ca^2+^ sparks with tetracaine does not increase the global [Ca^2+^]_i_ or constrict the SMA at 40 cmH_2_O. **a** Representative recordings of [Ca^2+^]_i_ changes from single smooth muscle cells from a pressurized (40 cmH_2_O) SMA in response to 100 nM Ibtx. **b** Average of normalized traces of [Ca^2+^]_i_ changes in the presence of Ibtx (65 cells). Traces in **a** and **b** were normalized as described in Methods. **c** Average trace of corresponding changes in vascular diameter in the presence of Ibtx (8 arteries). **d** Representative recordings of [Ca^2+^]_i_ changes from single smooth muscle cells from a pressurized (40 cmH_2_O) SMA in response to 100 μM Tet and 1 μM nifedipine (Nif). **e** Average of normalized traces of [Ca^2+^]_i_ changes at 40 cmH_2_O (26 cells). Traces in **d** and **e** were normalized as described in Methods. **f** Average trace of corresponding changes in vascular diameter (5 arteries) in the presence of Tet and Nif. Diameter changes were normalized to the average of values recorded between 30–60s (value indicated by ‘a’ was set to 1). [Ca^2+^]_i_ and diameter data were simultaneously acquired at 1 s intervals, however, for clarity, error bars (sem) are plotted only every 10s

**Fig. 4:**
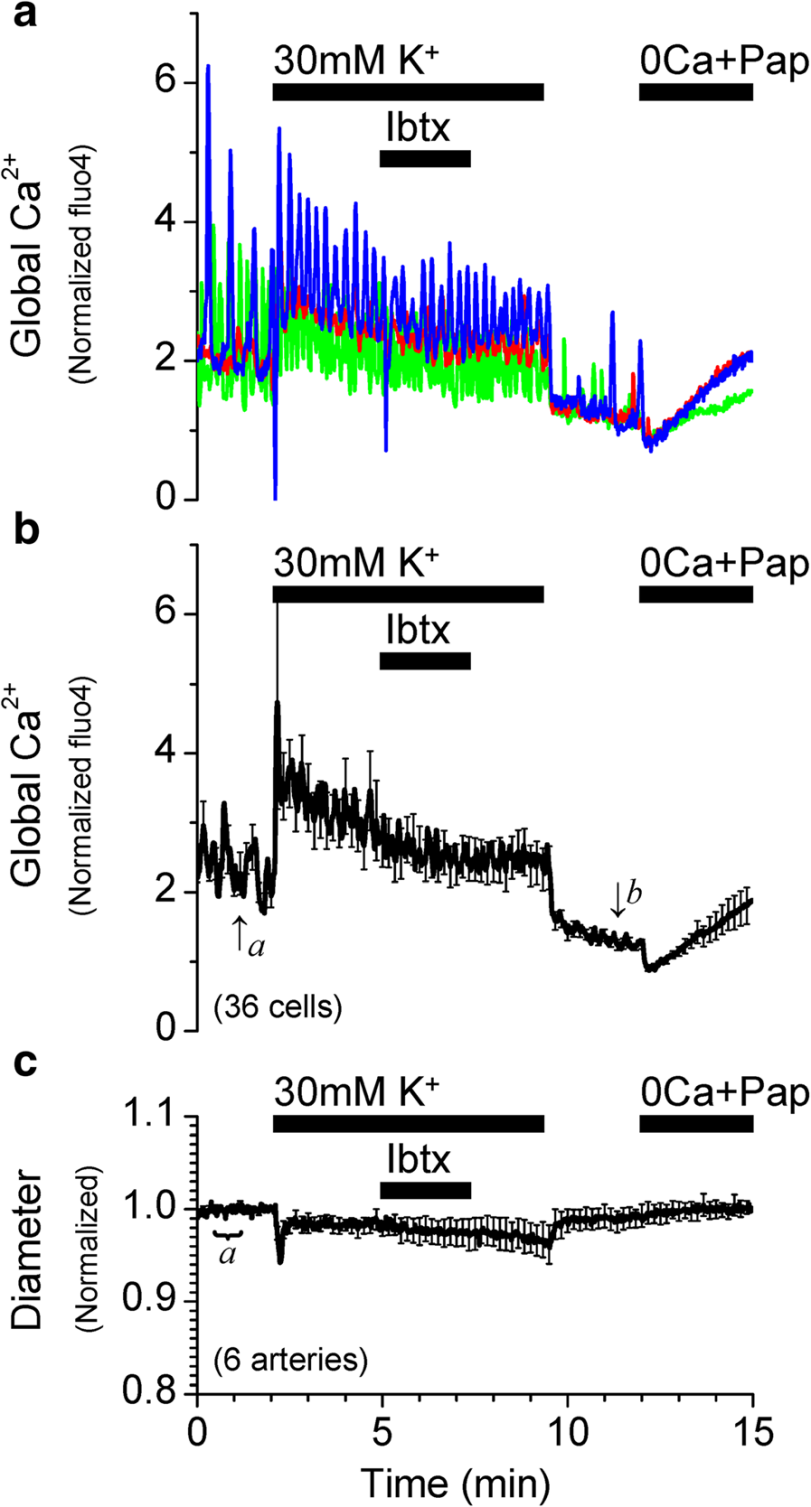
Inhibition of BK channels in the presence of high K^+^ does not increase the global [Ca^2+^]_i_ or constrict the SMA. **a** Representative recordings of [Ca^2+^]_i_ changes from single smooth muscle cells from a pressurized (40 cmH_2_O) SMA in the presence of PSS containing 30 mM K^+^ and 100 nM Ibtx. **b** Average of normalized traces of [Ca^2+^]_i_ changes in the presence of PSS containing 30 mM K^+^ and Ibtx (36 cells). Traces in **a** and **b** were normalized as described in Methods. **c** Average trace of corresponding changes in vascular diameter (6 arteries). Diameter changes were normalized to the average of values recorded between 30 – 60 s (value indicated by ‘a’ was set to 1). [Ca^2+^]_i_ and diameter data were simultaneously acquired at 1 s intervals, however, for clarity, error bars (sem) are plotted only every 10 s

**Fig. 5:**
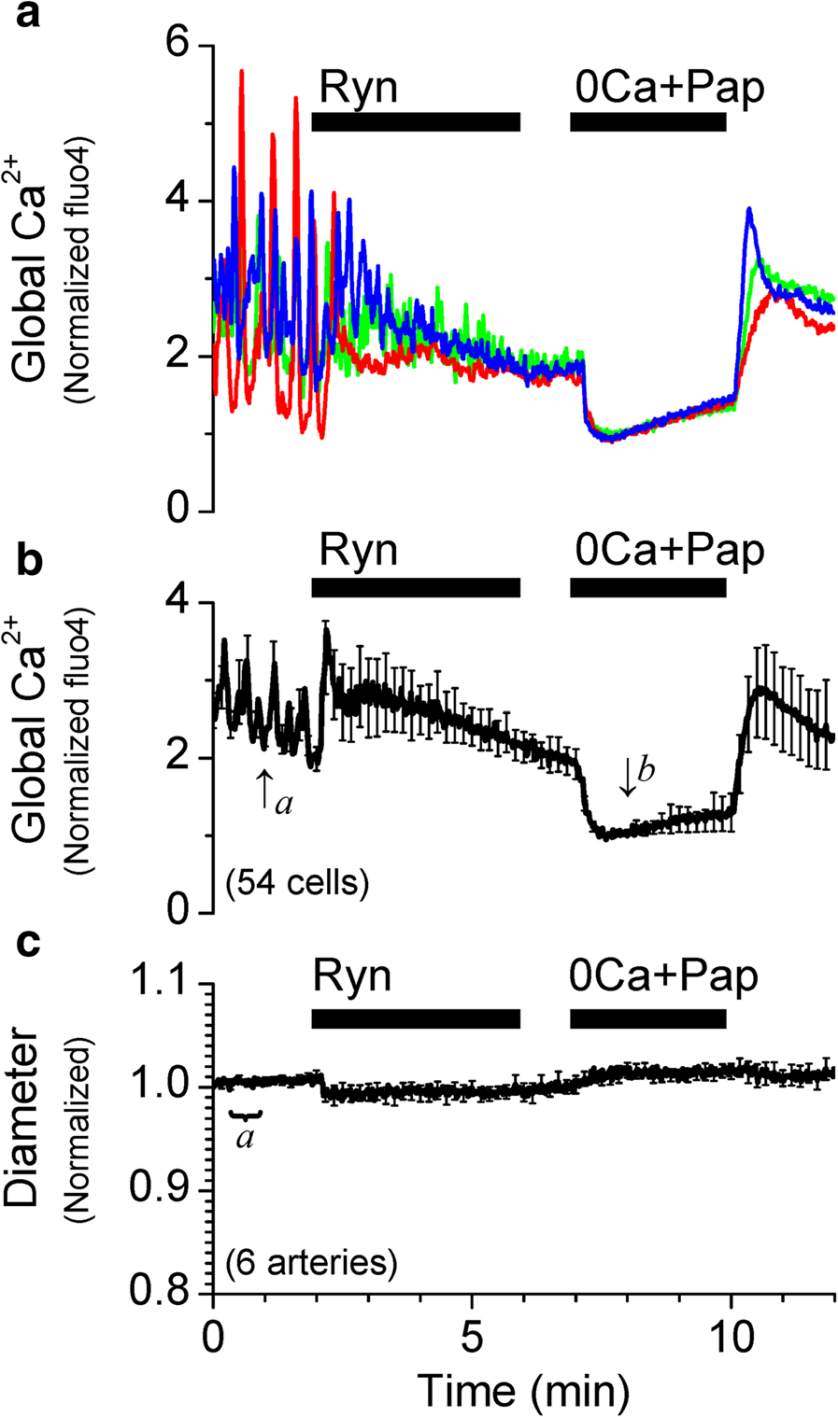
Inhibition of Ca^2+^ sparks with ryanodine results in a modest increase in global [Ca^2+^]_i_ but does not constrict the SMA at 60 cmH_2_O. **a** Representative recordings of [Ca^2+^]_i_ changes from single smooth muscle cells from a pressurized (60 cmH_2_O) SMA superfused with HEPES-buffered PSS in response to 10 μM Ryn. **b** Average of normalized traces of [Ca^2+^]_i_ changes at 60 cmH_2_O (54 cells). Traces in **a** and **b** were normalized as described in Methods. **c** Average trace of corresponding changes in vascular diameter (6 arteries). Diameter changes were normalized to the average of values recorded between 30 – 60 s (average value indicated by ‘a’ was set to 1). [Ca^2+^]_i_ and diameter data were simultaneously acquired at 1 s intervals, however, for clarity, error bars (sem) are plotted only every 10 s

In most arteries, inhibition of Ca^2+^ sparks and/or BK channels has been shown to increase SMC global Ca^2+^ to cause a robust vasoconstriction in a non-additive fashion, reflecting that Ca^2+^ sparks and BK channels are part of the same mechanism to hyperpolarize the membrane and limit Ca^2+^ influx, leading to vasorelaxation [17, 18, 20]. In the pressurized (40 cmH_2_O) SMA, application of 10 μM ryanodine inhibited Ca^2+^ sparks and appeared to similarly increase the average global cytosolic Ca^2+^ followed by a robust vasoconstriction (Fig. 2). However, application of 100 μM tetracaine, another known inhibitor of ryanodine receptors and Ca^2+^ sparks [24], or application of 100 nM iberiotoxin, a potent BK channel inhibitor, did not cause any change in global Ca^2+^ or vascular diameter similar to that produced by ryanodine (Fig. 3). Activation of BK channels is dependent on the local [Ca^2+^]_i_ as well as the membrane potential of the smooth muscle membrane [25]. It is possible that lack of an effect of iberiotoxin is a consequence of unopened BK channels caused by a hyperpolarized resting membrane potential in the smooth muscle cells of the SMA. To account for such a possibility, the pressurized SMA was superfused with PSS solution containing 30 mM K^+^. High K^+^ induced a transient increase in the global [Ca^2+^]_i_ and vasoconstriction. Under these conditions, 100 nM iberiotoxin remained without effect (Fig. 4). Furthermore, contrary to the effect at 40 cmH_2_O, 10 μM ryanodine increased global Ca^2+^ modestly and did not constrict the SMA pressurized at 60 cmH_2_O (Fig. 5), even though spark frequency is increased significantly from 40 to 60 cmH_2_O (Table 1).

These effects suggest that, unlike cerebral arteries, the ryanodine-induced increase in global Ca^2+^ and constriction at 40 cmH_2_O in the SMA may not be attributed to a loss of the hyperpolarizing influence of the Ca^2+^ spark-BK channel signaling mechanism. Under these conditions, ryanodine-sensitive Ca^2+^ sparks do not appear to regulate global Ca^2+^ and vascular tone via BK channels in the SMA. This raises the question as to the mechanism involved in the ryanodine-induced increase in the global Ca^2+^ and vasoconstriction. It is to be expected that at least a portion of the global Ca^2+^ is the result of Ca^2+^ influx via voltage-dependent Ca^2+^ channels (VDCCs), which are open at the physiological resting membrane potential in SMCs. Evidence for active VDCCs in the SMA comes from the observation of a decrease in global Ca^2+^ upon application of a reversible VDCC inhibitor, 2 μM nifedipine, in the presence of 100 μM tetracaine (Fig. 3d and e). It is possible that the remainder of the ryanodine-induced increase in the global Ca^2+^ is the result of ryanodine receptor-mediated Ca^2+^ release from the sarcoplasmic reticulum (SR). It is well-established that at a concentration of 10 μM, ryanodine binds to open ryanodine receptors and modifies the channel to lock them in an irreversible sub-conductance state of 234 pS [16, 26] that inhibits Ca^2+^ release and instead “leaks” SR Ca^2+^ into the cytosol. This is reflected in the transient increase in global Ca^2+^ immediately upon application of 10 μM ryanodine, followed by a slowly decaying plateau phase devoid of Ca^2+^ oscillations, indicating the relatively slow emptying of the SR through the partially open ryanodine receptors (Fig. 2a and b).

It is to be noted that the ryanodine-induced vasoconstriction continues to increase as the corresponding average global Ca^2+^ plateaus and then decreases (Fig. 2b and c). Indeed, the maximum vasoconstriction corresponds to the least increase in global Ca^2+^ induced by ryanodine, suggesting an increase in the Ca^2+^ sensitivity of constriction following the initial increase in the global Ca^2+^. In other words, the constriction induced by ryanodine at 40 cmH_2_O may be attributed to enhanced Ca^2+^ sensitivity of the SMA that is able to respond to the ryanodine-induced increase in intracellular Ca^2+^ with vasoconstriction. The observation that the ryanodine-induced constriction at 40 cmH_2_O is enhanced (Fig. 2c) compared to that at 60 cmH_2_O (Fig. 5c) suggests that the Ca^2+^ sensitivity at 40 cmH_2_O may be greater than at 60 cmH_2_O.

### Ca^2+^ sensitivity of the SMA decreases with increasing pressure

**Fig. 6:**
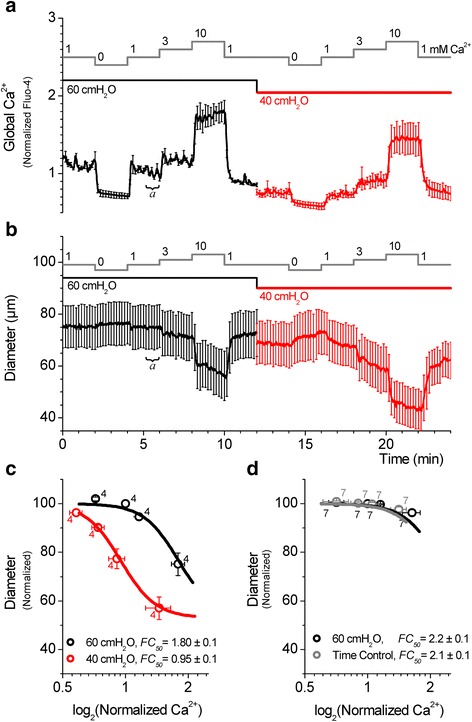
Ca^2+^ sensitivity of the SMA decreases with increase in pressure. **a**, **b** Vascular diameter and [Ca^2+^]_i_ changes in the presence of 0, 1, 3 and 10 mM Ca^2+^ were simultaneously measured from vessel segments pressurized at 60 cmH_2_O (black trace) followed by 40 cmH_2_O (*red trace*), as indicated. **a** Summary of changes in [Ca^2+^]_i_ measured as changes in fluorescence intensity. **b** Summary of corresponding diameter measurements first at 60 cmH_2_O and then at 40 cmH_2_O (4 arteries). Data were acquired at 1 s intervals, however, for clarity, error bars (sem) are plotted only every 10s. **c** Ca^2+^ sensitivity of SMA at 60 cmH_2_O (*black trace*) and 40 cmH_2_O (*red trace*). **d** Ca^2+^ sensitivity of SMA at 60 cmH_2_O (*black trace*) and time control at 60 cmH_2_O (*grey trace*). Numbers next to symbols represent the number of arteries. For **c** and **d**, average FC_50_ values are given as mean ± sem. Data points for normalized [Ca^2+^]_i_ and diameter were obtained by averaging diameter and fluorescence intensity measurements over the last 30 s of each Ca^2+^ step and normalizing these values against the average value obtained in PSS containing 1 mM Ca^2+^ at 60 cmH_2_O (denoted as ‘a’)

The Ca^2+^ sensitivity at 40 and 60 cmH_2_O was determined from simultaneous measurements of the cytosolic Ca^2+^ and the vascular diameter. The cytosolic Ca^2+^ concentration was manipulated by altering the Ca^2+^ concentration in the superfusate (Fig. 6a and b). Normalized cytosolic Ca^2+^ and corresponding vascular diameter measurements were plotted against each other and fitted to the Hill equation. A decrease in the pressure from 60 to 40 cmH_2_O shifted the Ca^2+^-diameter relationship to the left on the Ca^2+^ axis, indicating a dramatic increase in the Ca^2+^ sensitivity, with nearly 2-fold decrease in the Ca^2+^ required for a half-maximal constriction at 40 cmH_2_O compared to that at 60 cmH_2_O (Fig. 6c), whereas a time control repeated at 60 cmH_2_O did not (Fig. 6d). Thus, the modest increase in global Ca^2+^ caused by ryanodine at 60 cmH_2_O was insufficient to constrict the SMA at this pressure, whereas the enhanced Ca^2+^ sensitivity at 40 cmH_2_O allowed for a robust constriction for an increase in global Ca^2+^.

### Endothelin enhances the ryanodine-induced vasoconstriction

**Fig. 7:**
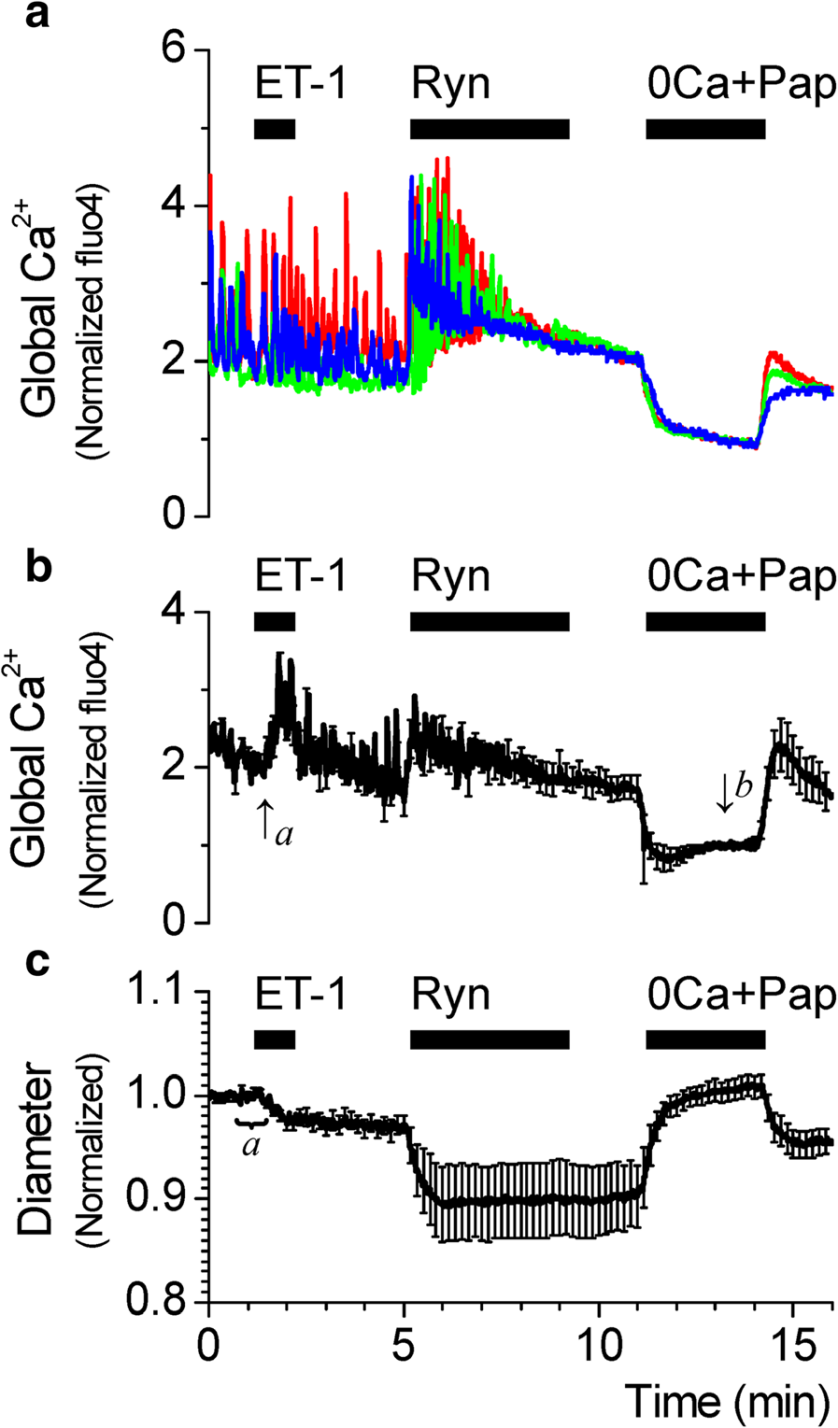
Inhibition of Ca^2+^ sparks with ryanodine increases global Ca^2+^ and constricts SMA at 60 cmH_2_O following an increase in Ca^2+^ sensitivity by endothelin-1. **a** Representative recordings of cytosolic Ca^2+^ changes from single smooth muscle cells from a SMA loaded with fluorescent dye fluo4 and pressurized to 60 cmH2O in response to 10 μM ryanodine after treatment with 1 nM endothelin-1 (ET-1). **b** Average of normalized traces of cytosolic Ca^2+^ changes at 60 cmH_2_O (64 cells from 7 arteries). Traces in **a** and **b** were normalized as described in Methods. **c** Average trace of corresponding changes in vascular diameter of SMA pressurized at 60 cmH_2_O (15 arteries). Diameter changes were normalized to the average of values recorded between 30–60 s (value indicated by ‘a’ was set to 1). Ca^2+^ and diameter data were simultaneously acquired at 1 s intervals, however, for clarity, error bars (sem) are plotted only every 10 s

The result above implies that conditions that increase the Ca^2+^ sensitivity at 60 cmH_2_O would increase the ryanodine-induced vasoconstriction. Consequently, the SMA pressurized at 60 cmH_2_O was first exposed to 1 nM endothelin-1 (ET-1) for 1 min. It has been previously shown that endothelin-1 acts via ET_A_ receptors to increase the Ca^2+^ sensitivity of the SMA in a rho-kinase dependent manner [27]. ET-1 caused a transient increase in the cytosolic Ca^2+^ concentration and a persistent vasoconstriction, consistent with an increase in the Ca^2+^ sensitivity (Fig. 7). Under these conditions, 10 μM ryanodine caused a vasoconstriction that was enhanced compared to that observed in the absence of ET-1 (Fig. 5). These results support the concept that ryanodine increases global Ca^2+^ and constricts the SMA when the Ca^2+^ sensitivity is high. Ca^2+^ sensitivity of SMC contraction is hence a critical factor in the regulation of the vascular diameter of the SMA in response to changes in pressure and cytosolic global Ca^2+^.

## Discussion

Salient findings of the present study are 1) Ca^2+^ spark frequency in the pressurized SMA increases with pressure; 2) Inhibition of Ca^2+^ sparks with ryanodine increases global Ca^2+^ and causes a robust vasoconstriction, however, ryanodine-induced effects on global Ca^2+^ and vascular diameter are not reproduced by other inhibitors of Ca^2+^ sparks or by inhibitors of BK channels as would be expected if Ca^2+^ sparks activated BK channels to regulate membrane potential, global Ca^2+^ and vascular diameter. 3) The ryanodine-induced vasoconstrictions depends on the Ca^2+^ sensitivity, which is higher at 40 cmH_2_O compared to that at 60 cmH_2_O and can be enhanced with endothelin-1.

### Ca^2+^ sparks

Ca^2+^ sparks in the pressurized SMA occurred with a lower frequency than in unpressurized SMA [22], but with a similar frequency, spatial width and spark site density as observed in smooth muscle cells of cerebral pial arteries [15, 19, 28] and pressurized mesenteric arteries [21, 29]. The increase in Ca^2+^ spark frequency in response to a 20 cmH_2_O (~ 14 mmHg) increase in pressure, is also consistent with observations made in cerebral arteries [28]. However, as in unpressurized SMA, the time of half decay of Ca^2+^ sparks was far shorter (~17–19 ms) than that observed for Ca^2+^ sparks in cerebral arteries, but closer to that found in rat heart [16, 30]. Ca^2+^ spark amplitudes and decay times are generally a reflection of the number as well as the isoform of ryanodine receptors (RyRs) present in a spark cluster. Typically, Ca^2+^ spark sites are composed of 4 – 6 RyRs, giving a punctate staining pattern in immunolocalization studies. In the SMA, the distribution pattern of RyRs in SMCs shows a uniform expression throughout the SR rather than a punctate expression expected of ryanodine receptors clustered in spark sites [22] and may underlie the observed differences in the temporal properties and functional role of Ca^2+^ sparks in the SMA.

### Absence of the Ca^2+^ spark/BK channel hyperpolarizing mechanism in the SMA

The Ca^2+^ spark/BK channel signaling complex provides an important vasodilatory mechanism in preventing or mitigating pressure- or agonist-induced vasoconstrictions in arteries. This negative feedback mechanism in regulating vascular tone is evident from observations that pharmacological inhibition of Ca^2+^ sparks and/or BK channels or SMC-specific genetic manipulation of BK channel or RyR expression leads to the loss of this hyperpolarizing signal leading to membrane depolarization, increased VDCC activation, increase in Ca^2+^ influx and global Ca^2+^ and enhanced vasoconstriction [18, 20, 31–33]. However, Ca^2+^ sparks have not always been linked to a hyperpolarizing or vasodilatory mechanism. Other studies have observed excitatory roles for Ca^2+^ sparks and RyR-mediated Ca^2+^ release in small diameter arterioles. Kur et al. [24] reported that, contrary to the conventional hyperpolarizing mechanism, Ca^2+^ sparks in retinal arterioles combined to form Ca^2+^ waves and enhanced the myogenic tone. Westcott et al. [34] reported SMCs of murine cremaster muscle feed arterioles to express diffused staining of RyRs without manifesting Ca^2+^ sparks and no coupling with BK channels, while SMCs of upstream feed arteries exhibited clustered staining of RyRs, robust Ca^2+^ sparks and spatial and functional coupling to BK channels, indicating heterogeneity of RyR function within the same vascular tree. In the SMA, the effects of ryanodine on global Ca^2+^ and vascular diameter at 40 cmH_2_O seemed to suggest, at first, a vasodilatory mechanism for Ca^2+^ sparks acting via BK channels. However, the failure of tetracaine, an RyR inhibitor, which inhibits Ca^2+^ sparks without depleting the SR, and iberiotoxin, a BK channel inhibitor, to produce similar effects on global Ca^2+^ and diameter as ryanodine (Figs. 2 and 3) combined with the non-effect of BK channel inhibition following membrane depolarization by external application of high K^+^ (Fig. 4) or increase in pressure (Fig. 5) disproves the regulation of vascular tone of the SMA by the Ca^2+^ spark/BK channel mechanism.

### Regulation of vascular tone by Ca^2+^ sensitivity

Changes in SMC global Ca^2+^ have generally been accepted as the central mechanism regulating SMC contractility in the development of pressure-dependent myogenic tone [35]. More recently, the contribution of Ca^2+^-independent processes that regulate the Ca^2+^ sensitivity of the myofilament in the development of myogenic tone have been better described [36]. In cerebral and skeletal resistance arteries, increases in intravascular pressure are associated with increases in the Ca^2+^ sensitivity achieved by balancing the relative activities of myosin light chain kinase and myosin light chain phosphatase in a PKC and rho-kinase-dependent manner. Such changes in Ca^2+^ sensitivity further augment the Ca^2+^-dependent myogenic vasoconstrictions [36–40]. We have previously shown that myogenic tone in the male, but not female, gerbil SMA is regulated not by changes in the SMC global Ca^2+^ but by rho-kinase-mediated changes in the Ca^2+^ sensitivity of contraction, which was revealed under inhibition of NO-mediated signaling [23]. The present study shows that the regulation of vascular tone in the female gerbil SMA is also determined by the Ca^2+^ sensitivity of the myofilament, with the crucial difference that increase in intravascular pressure significantly lowered the Ca^2+^ sensitivity (Fig. 7). This finding is consistent with the development of small myogenic tones with increasing intravascular pressures in the SMA [12]. Rho-kinase-dependent regulation of Ca^2+^ sensitivity also plays a significant role in mediating the vascular effects of endogenous vasoconstrictors and agonists [41–43]. Further studies are required to elucidate the mechanisms underlying the relationship between pressure and Ca^2+^sensitivity in the SMA.

## Conclusions

In conclusion, in this study, we have shown in the gerbil spiral modiolar artery that Ca^2+^ sparks, while present, do not regulate vascular tone and global Ca^2+^ by activating BK channels. Instead, ryanodine-receptor mediated increases in global Ca^2+^ and vasoconstriction depend on the Ca^2+^ sensitivity of SMC contraction, which is enhanced at lower pressures or by regulating rho-kinase activity.

It remains to be seen whether such mechanisms of vascular tone regulation as described in this study are applicable to spiral modiolar arteries in general or particularly unique to the gerbil spiral modiolar artery. Gerbils are commonly favored over other rodents such as mice and rats as hearing models for investigations into the causes for age-related hearing loss involving pathological changes in both peripheral and central auditory system components. Gerbils are uniquely suited for such investigations as they exhibit sensitive hearing in the low frequency ranges (below 4 kHz) that are relevant for human auditory perception, compared to the much higher thresholds in mice and rats for the same frequency range [44]. Thus, the differences observed in the regulation of the gerbil SMA by Ca^2+^ sparks and BK channels compared to the observations made in arteries from other extensively studied rodent species become relevant in the choice of appropriate models for future interpretation of hearing studies and pharmacological interventions.
